# Factors affecting time to publication in information science

**DOI:** 10.1007/s11192-022-04296-8

**Published:** 2022-02-27

**Authors:** Zehra Taşkın, Abdülkadir Taşkın, Güleda Doğan, Emanuel Kulczycki

**Affiliations:** 1 Scholarly Communication Research Group, Adam Mickiewicz University, Poznań, Poland; 2 Department of Information Management, Hacettepe University, Ankara, Turkey; 3 Mantis Software Company, Ankara, Turkey

**Keywords:** Publication time, Time to publication, Scholarly communication, Publication delay, Information science

## Abstract

Publication speed is one of the important aspects of scholarly communication since various research performance evaluation systems are based mostly on published papers. This study aims to reveal the factors affecting the publication speed of journals. In this context, six information science journals: *ASLIB Journal of Information Management, Journal of Documentation, Journal of Informetrics, Journal of the Association for Information Science and Technology, Online Information Review,* and *Scientometrics* are analysed in terms of time to publication (from submission to decision). Our results show that publication time is significantly shorter when an editorial board member or a productive author of a given journal is one of the authors, in compare with the articles. submitted by other authors. The number of authors has a time-prolonging effect on publication time, as expected. On the other hand, publications with more citations were accepted in a shorter time. The papers with authors from central countries and high-income countries have an advantage of shorter publication time. Thus, this study shows that researchers who publish papers with popular and successful researchers from central countries have the advantage of the speed of publication which may have substantial effects on the future academic work, especially of early career researchers.

## Introduction

Scholarly communication is defined as the “system through which research and other scholarly writings are created, evaluated for quality, disseminated to the scholarly community, and preserved for future use” (ACRL, 2006). As the definition refers, scholarly communication is not limited to the communicative activity of scholars. It also covers groups who shared propensity for communicative activity such as peer-reviewers, editors, indexers, information seekers, and readers (Borgman & Furner, 2002). The process of publishing scientific studies is the result of the first three actors working together. While scholarly communication was slow in the past because of the traditional publication practices based on print journals, it is known that these processes have been accelerated with the advancement of technology and the shift of journal publishing to the electronic environment (Lyman, 2013). In 1969, the factors influencing publication time-lag identified as author-based problems (not meeting the deadlines or templates etc.), heavy workloads of editorial board members, priority for publication, length of the papers, and costs (Jain & Goyal, 1969). With the transition of all publishing processes to electronic media, the spread of open access and the diversification of publishing platforms, it was predicted that traditional scholarly communication tools would be improved (ACRL, 2006; Odlyzko, 2002; Rowlands et al., 2004). During that time, the only concern was information overload, but, Odlyzko, (2002) indicated that these concerns were exaggerated because people could find their way to the serious information sources. However, at the point reached today, information overload is the most important problem of electronic publishing, and unfortunately, whose solution has not been found yet. The workload of all the actors of scholarly communication, especially the reviewers, which is created by the aforementioned information overload to some extent and thus the lack of time makes all scholarly communication processes decelerate.

Today, there is a publishing ecosystem that is growing much faster than anticipated. Many more publications are expected in the future (Taşkın, 2021a, 2021b). Publish or perish culture encourages scientists to produce outputs as soon as possible rather than conducting meticulous research (Williams, 2021). Editors experience difficulties to find peer-reviewers and getting reviewers’ acceptance. Besides, reviewers’ response times are long and the reviews are almost never of equal quality by nature (Tenorio-Fornés & Tirador, 2020). On the other hand, the coronavirus has deeply affected the scholarly communication processes. Guinart and de Filippis (2021) called this phenomenon “the publication fever”. This fever has reduced the time to publication of coronavirus-related papers, while the others’ time-lags are getting longer (Behera et al., 2021; Reiss, 2021).

Various studies on scholarly communication points out that not all the papers have equal opportunities in terms of time to publication. There are underrepresented groups of researchers that have disadvantages (based on age, title, geography, native language, subject etc.) (Valoyes-Chávez et al., 2021). While some researchers’ articles are published in a very short time, this process may take longer for some other researchers. However, publication speed has importance for all researchers who seek tenure and secure work conditions. Long review processes, the lack of standardization in the editorial control, and differences from discipline to discipline cause researchers to have negative opinions about the journals (Huisman & Smits, 2017).

The main aim of this paper is to reveal factors affecting the time to publication for information science (IS) journals. Thus, the research questions are:

1. Do the types of peer review affect the time to publication?
2. Does being an editorial board member of a journal shorten time to publication?
3. Does being one of the top-ten authors of the journal shorten time to publication?
4. Does having more than one role in a journal affect time to publication?
5. What is the difference between time to publication for central researchers (top-ten authors and editorial board members) in the IS field and others?
6. Does the number of authors affect the time to publication?
7. How do the authors’ countries and their income levels affect time to publication?
8. Is there a significant relationship between time to publication and the number of citations after the publication?

## Literature review

**Fig. 1:**
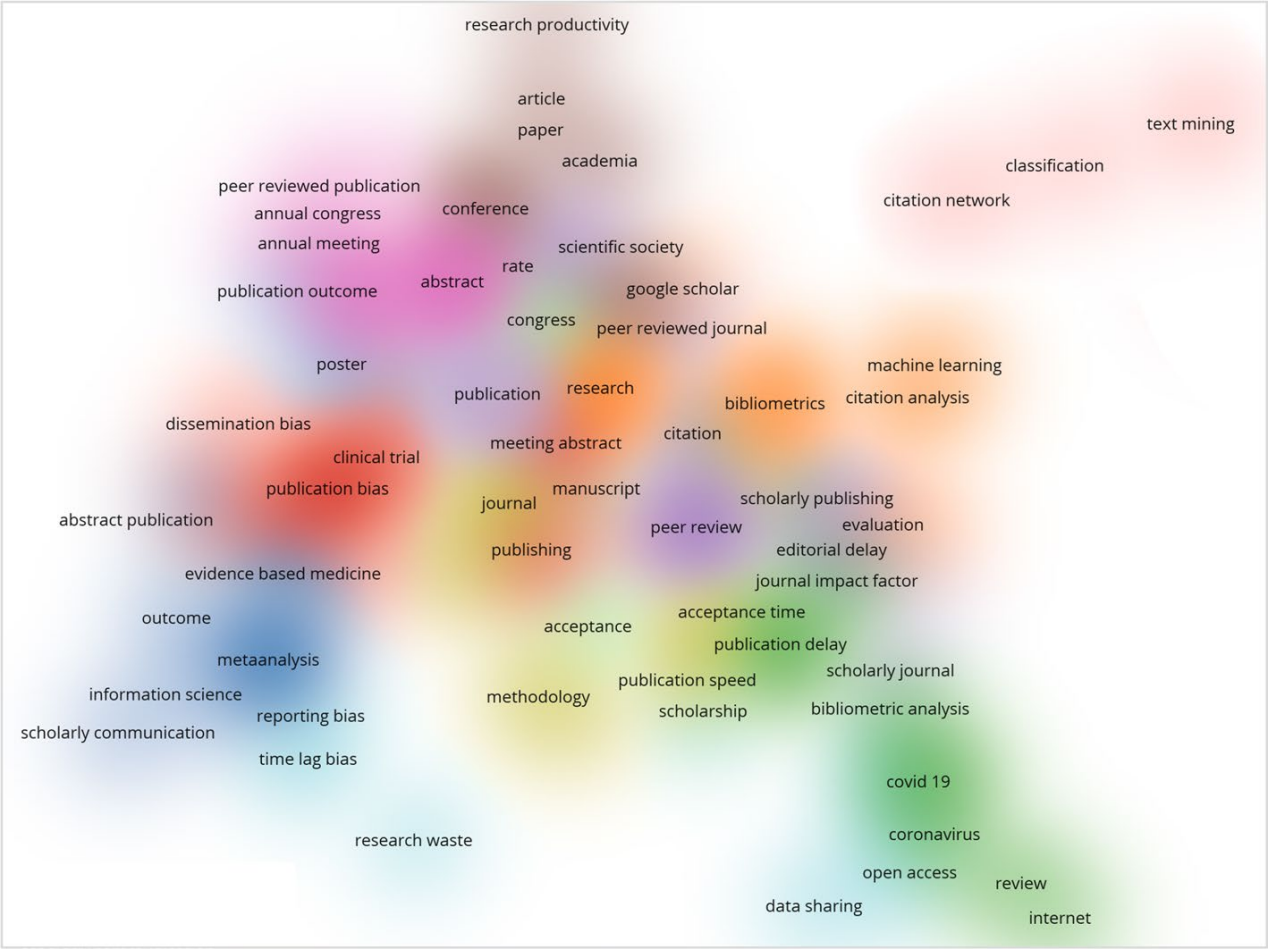
Literature studies on time to publication

When the articles in Web of Science on publication time were investigated in terms of author keywords, this subject has been examined in many different dimensions from peer-review to publication bias (see Fig. 1).^1^ The core theme of these studies is publication speed during peer-review. Also, the network shows that time-lags are often considered as an issue of bias.

The main purpose of many studies investigating peer-review time in the literature has been to reveal why some articles are accepted very quickly, while others have very long processes (e.g. Bilalli et al., 2021; Kljaković-Gašpić et al., 2003; Mrowinski et al., 2016). According to Mrowinski et al. (2016), the relationship between the editor and reviewer determines the completion rate and peer-review time. Known reviewers reply review requests more quickly than unknown reviewers. Besides, Kljaković-Gašpić et al. (2003) indicated that reviewers of small journals took their responsibility seriously and responded more quickly in comparison with big publishers’ big journals. Sabaj et al. (2015) revealed that partial agreement between reviewers was related to longer publication times for university journals in Chile. Also, it is proved that peer-review time differs in terms of disciplines (Huisman & Smits, 2017; Sabaj et al., 2015).

In addition to differences in peer-review time, it has always been important to understand how long is too long for peer-review (Nguyen et al., 2015). While the authors’ average publication time expectation was 6 weeks, the time they experienced was 14 weeks. It creates negative consequences and a negative impact on the author morale. On the other hand, a statistical decrease was identified from 1980 to 2012 because of changes in publication formats (from paper to online) (Lyman, 2013). However, it seems that this decrease has not met the publication speed expectations of authors.

In 2016, it was emphasized that the speed of peer-review is of great importance in some subjects where timeliness is important (Cooke et al., 2016). The accuracy of this assessment has been proven after the publication explosion which occurred after the COVID-19 pandemic. The peer-review process of articles published on the pandemic takes a shorter time than the others, however, problems related to the quality of peer-review reports have arisen (Horbach, 2021). It has also been revealed that the increased workload of the peers after the pandemic affected the peer-review times and all the scholarly communication processes negatively (Oh, 2020; Waltman et al., 2021). As stated in the Introduction part, difficulties in finding reviewers are one of the factors that prolong the publication time of the articles. According to Tite and Schroter (2007), the most used reason to decline to review is the workload of researchers. Also, financial and non-financial (free subscription or acknowledge) incentives are not enough to encourage reviewers to peer-review.

Various suggestions have been developed in the literature to minimize the problems related to peer-review time. They are: (1) Using new generation peer-review models such as real-time comments and post-publication peer-review (Swanson et al., 2012; Teixeira da Silva & Dobránszki, 2015), (2) Incentivizing peer-reviewers (Hauser & Fehr, 2007; Nguyen et al., 2015), (3) Paying for the peer-review service (financial or otherwise) (Kumar, 2014) and (4) Providing a balance between authors and peer-reviewers (by editors) (Huisman & Smits, 2017).

In addition to review time-related factors affecting time to publication, there are various reasons behind publication speed, such as pre-publication bias or editorial delay (Yegros & Amat, 2009). For example, the publication time of papers with negative results is quite longer than the papers with positive results (Stern & Simes, 1997). Editorial delays can be affected by authors’ experiences (Yegros & Amat, 2009), and these short-delayed papers written by well-known authors can point to solicited papers that are almost immediately accepted by the editor-in-chief (Shen et al., 2015). However, it can provide a cumulative advantage to known researchers and disadvantages to others. An important step to reduce pre-publication bias is to provide open and transparent journal operations (Stamm et al., 2007). Scholarly communication is a long process that covers many actors in it. Our article aims to reveal publication time differences for papers and the possible reasons behind them. It is important to understand these factors to enhance scholarly communication processes.

## Data and methods

To achieve the aim of the study, three single-blind journals of information science (IS) field (*Journal of Informetrics* [JOI],^2^*Journal of the Association for Information Science and Technology* [JASIST]^3^ and *Scientometrics* [SCIM]^4^), and three double-blind journals (*ASLIB Journal of Information Management* [ASLIB],^5^*Journal of Documentation* [JDOC]^6^*and Online Information Review* [OIR]^7^) that provide submission and decision dates were chosen. A total of 3,816 articles that were published between 2016 and 2020 were evaluated deeply. Metadata of articles were gathered from Web of Science on January 2, 2021. In the dataset, a total of 2843 single and 973 double-blind articles were stored. 45% (1715) of these articles were published in *SCIM*, 20% (753) in *JASIST*, 10% in *JDOC* (381), *JOI* (375), and *OIR* (367), and 5% (225) in *ASLIB*. The main limitation of our study is the volume and subject differences between selected journals. While *SCIM* publishes 12 issues and almost 350 articles per year, the average number of articles for other journals are 156 for *JASIST*, 76 for *JDOC*, 75 for *JOI*, 73 for *OIR* and 45 for *ASLIB*. Also, the selected journals serve different sub-fields of information science (Taşkın, 2021a, 2021b) and it makes the comparison difficult. To handle this problem, we provided statistics for each journal separately. It is important to note that our paper aims to draw a frame for the whole information science field. However, further studies are needed to understand the editorial processing time of each sub-field.

To collect the names of editorial board members, the websites of journals were used. All members of the editorial boards were considered. However, considering the important editorial board change for *JOI* in 2019 (Larivière, 2019), two different lists were used for this journal. One for the publications before 2019 and one for the publications after 2019.

To answer the third, fourth and fifth research questions, the top-ten productive authors of each journal in terms of their publication counts were determined. Because of the differences in the number of authors that the journals have, a journal-based assessment was presented besides the general assessment. For finding the top-ten of each journal, six different searches were done in Web of Science. The last 10 years (2011–2020) were considered. Only articles and reviews were covered. If the tenth and eleventh authors had the same number of publications, two of them were added to the dataset. After finding the top-ten authors in Web of Science, the articles were classified accordingly.

Number of authors were grouped in two different ways. Seven groups for general analysis (1, 2, 3, 4, 5, 6, 7+ authors) and four groups for journal-based analysis (1, 2, 3–5, 6+ authors) because of the frequencies. Citations per year after publication/acceptance were classified into five groups (0, 0.17–2, 2.17–4, 4.20–7, 7.20+ citations per year) considering the citation frequencies of the publications. In addition, World Bank’s country and lending groups were used for the classification of affiliation countries of authors (World Bank Country & Lending Groups – World Bank Data Help Desk, 2020). Although it is possible to calculate publication times for collaborative papers (different income levels or geography) using the fractional counting method, the results for each collaboration pattern (for example the differences between publication times of high-low income collaboration and high-upper income collaboration) are important for our study. Therefore, we used the full counting method for presentation.

The codes were written using Python to get publications’ timeline data automatically from journal websites (Taşkın, 2021a, 2021b). After getting the timeline data from journal websites, the publication time for each article was calculated. In our study, we defined the “publication time” as the number of days from submission to decision.

Although we aimed to conduct a regression analysis to evaluate the effects of all the abovementioned factors together, the data didn’t meet the assumptions. To compare the publication times in terms of the journal, peer review types, the number of authors, country group and income, and citation per year Kruskal Wallis, Mann Whitney, and *χ*^2^ tests were applied considering the assumptions required to apply parametric testing are not met. Effect sizes were also calculated for the positive test results. Formula (1) shows the effect size calculation for Kruskal Wallis ($\eta_{H}^{2} )$, and Formula (2) for Mann Whitney U $(r_{G})$ tests where *H* is the Kruskal–Wallis test statistic, *k* is the number of groups, $\overline{R}_{A}$ and $\overline{R}_{B}$ are the average ranks for groups, *n* and $N_{T}$ is the total number of observations (Cohen, 2013, pp. 10–11, 19–20).

1 $$\eta_{H}^{2} = \left( {H - k + 1} \right)/\left( {n - k} \right)$$

2 $$r_{G} = 2\left( {\overline{R}_{A} - \overline{R}_{B} } \right)/N_{T}$$

We used SPSS (version 21) and RCommander for statistical tests and descriptives; RCommander (with KMggplot2 plugin) and Flourish Studio for visualization.

## Findings

### Effects of single- and double-blind reviews on publication time

**Fig. 2:**
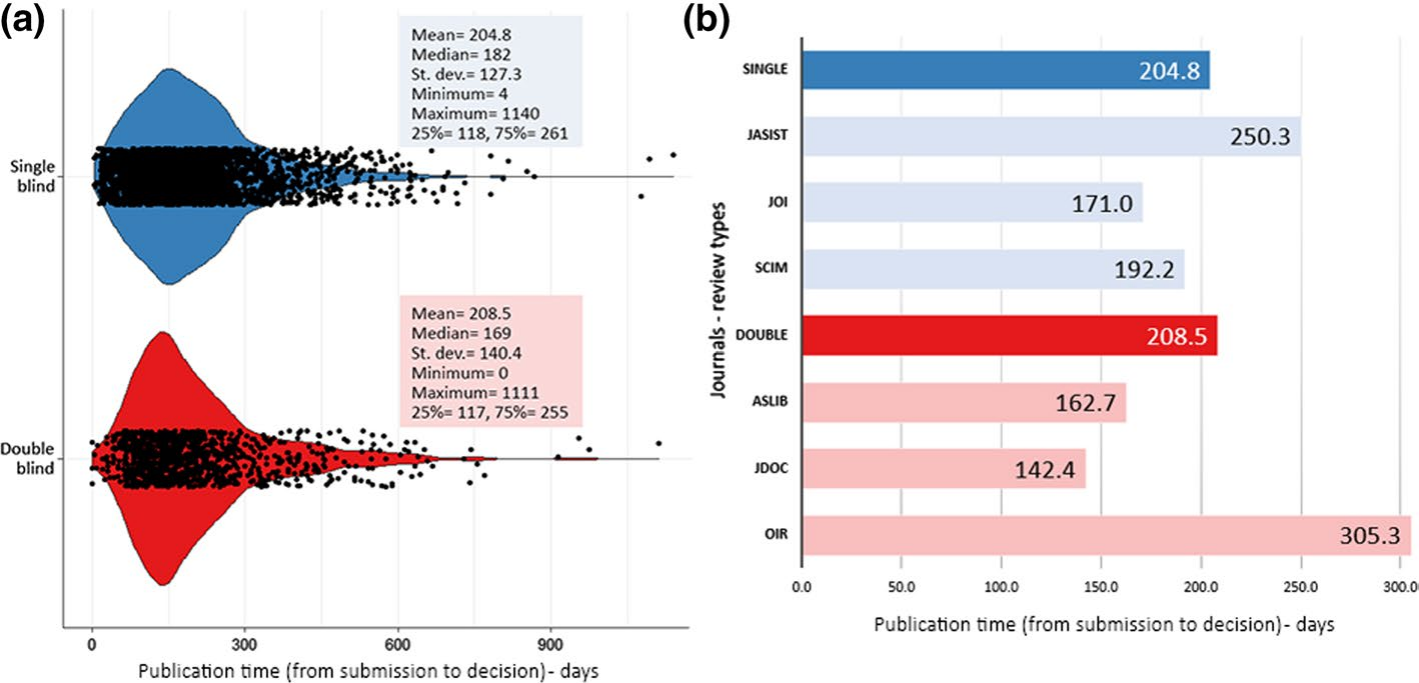
Publication time differences regarding peer review types

When it was investigated whether the type of peer review affects the time to publication, small differences were found between the two groups (see Fig. 2). Although the acceptance periods of the specific journals varied, the average time to publication of the single- and double-blind review types were very similar. Mann–Whitney *U* test confirmed this similarity (*U* = 1,358,685.500, *Z* = − 0.824, *p* = 0.410). On the other hand, significant differences were found between the journals’ publication time regardless of peer review type. According to the results of the Kruskal Wallis test, significant differences were found for the average publication time (*H* = 401.315, *p* < 0.001, $\eta_{H}^{2}$ = 0.104). When all journals were compared to understand the source of differences, there were no statistically significant differences found between *ASLIB* & *JOI* and *JDOC* & *JOI* at %99.9 confidence level.

### Effects of being an editorial board member on publication time

**Fig. 3:**
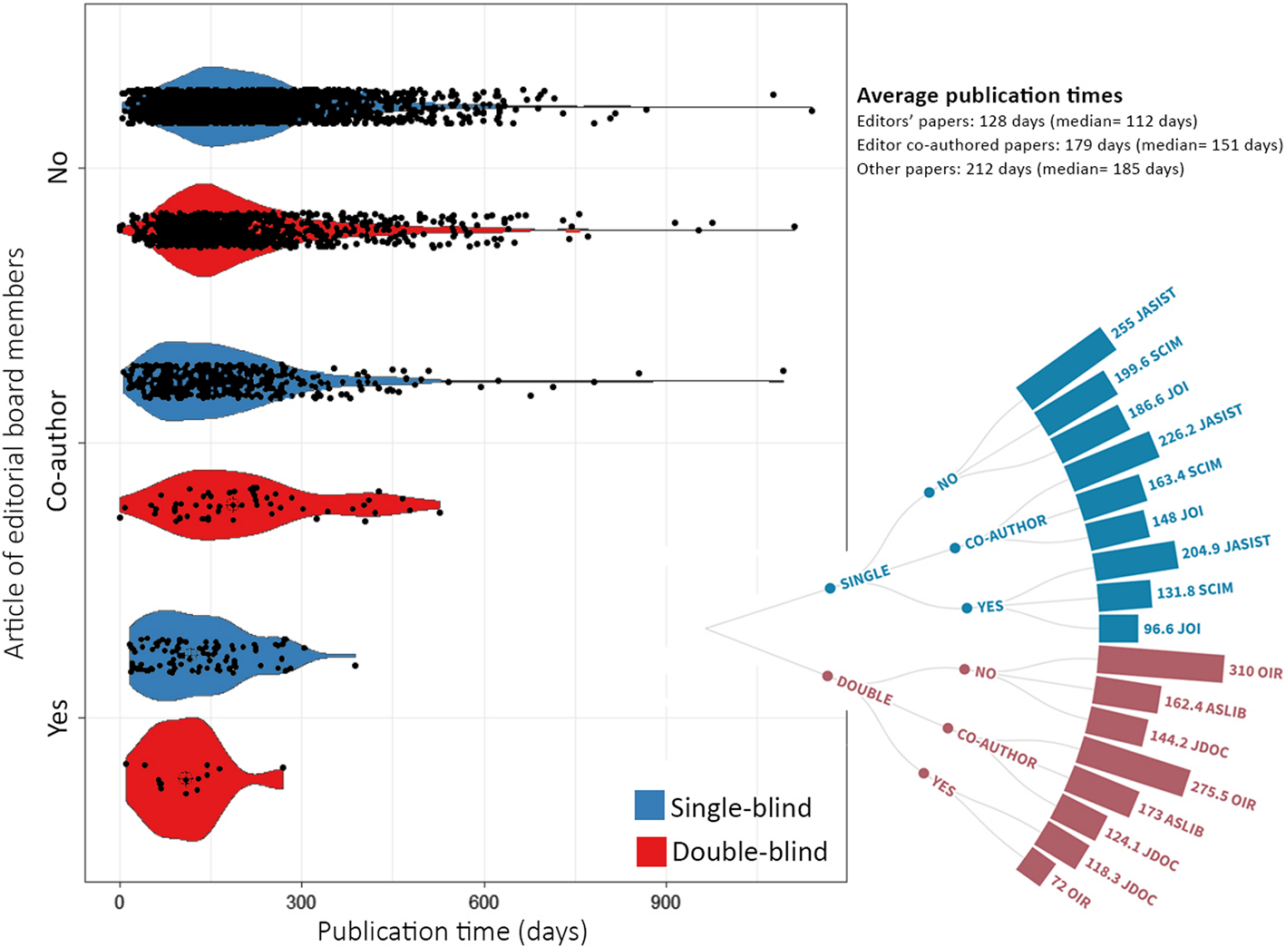
Publication times of papers written by editorial board members

Being an editorial board member is important in showing that researchers have achieved a certain scientific level, have proven their scientific merit in the field, and thereby have become a decision-maker for a journal (Bedeian et al., 2009; Pardeck & Meinert, 1999). Members of the editorial boards are often selected among the most popular researchers in their fields with high scientific competencies. Therefore, it is expected that the publication time of the articles written by editorial board members may be completed faster in parallel with the recognition, experience, and popularity in the field. The differences between the publication time of editorial board members’ and other authors’ papers are statistically significant (*H* = 92.892, *p* < 0.001, $\eta_{H}^{2}$ = 0.024).^8^ Figure 3 shows the publication times of papers written by editorial board members and time differences between the journals. Each black dot in the violin plot indicates a single paper. According to the results, when an editorial board member is a co-author of a study, it makes the publication process shorter. It confirms a recent study (Zhang et al., 2021) that reveals the positive effects of editorial boards’ cooperation with the authors’ publications. Furthermore, this difference is more obvious for single-blind journals ($\eta_{H}^{2}$ = 0.032).

### Papers from the top-ten authors of the journals

**Fig. 4:**
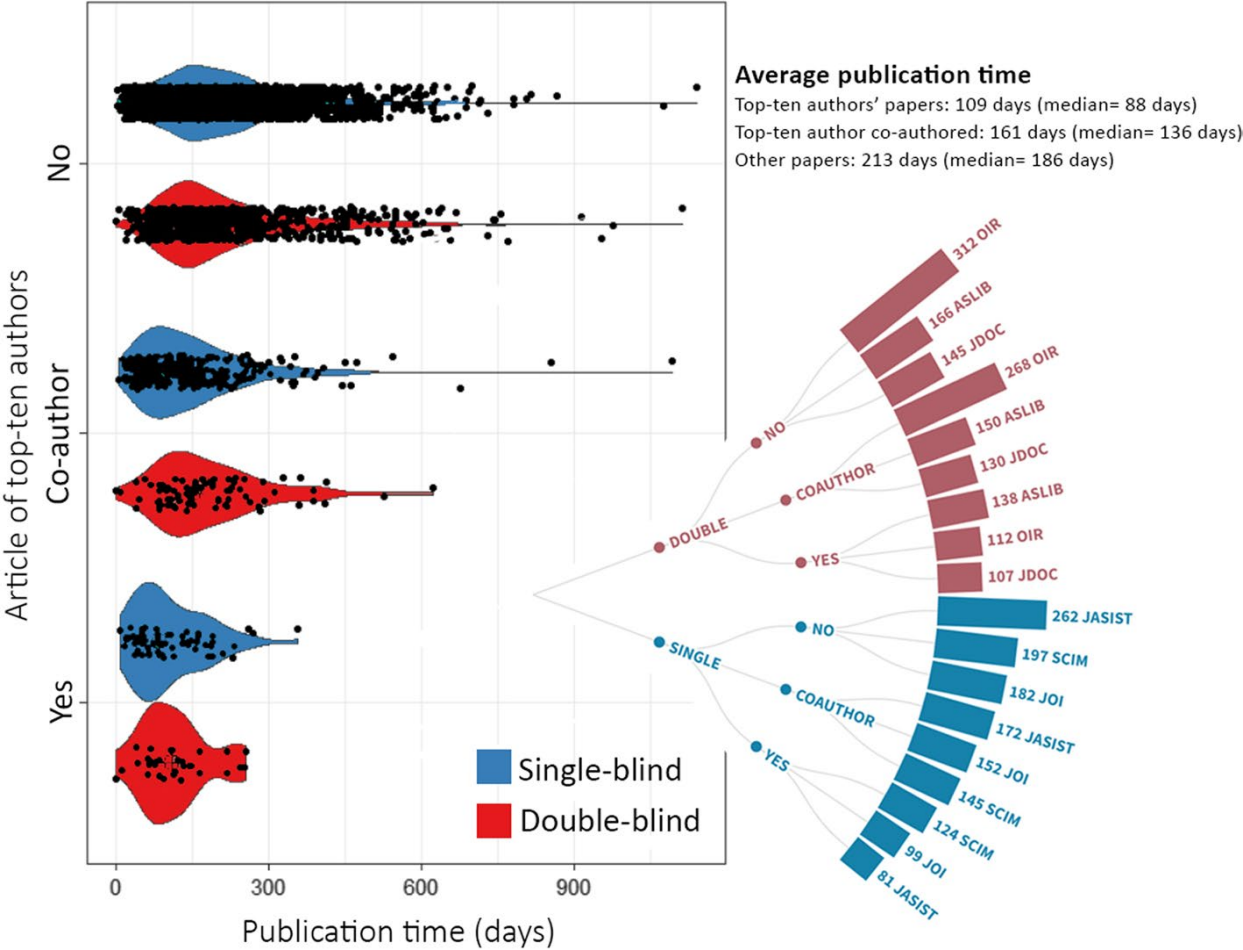
Publication time of papers written by top-ten authors of the journals

According to Merton (1968, p. 61), the works of scientists who have an outstanding position in science have been validated by judgments of the average quality of their past work. Therefore, it is easier to accept the works of outstanding authors by the journals. To confirm whether this approach affects the review durations, the publication times of the works from the top-ten authors of each journal were evaluated in this study. Figure 4 shows the publication times of papers written by top-ten authors and the differences between the journals. Each black dot in the violin plot indicates a single paper. The distribution is quite similar to editorial boards’ papers.

The results show that there are significant differences in publication time for the papers from top-ten authors and others (*H*(2) = 151.477, *p* < 0.001, $\eta_{H}^{2}$ = 0.039). The effects sizes calculated for each of the three pairs showed that the most significant difference is between the papers of top-ten authors (Yes in Fig. 4) and papers with no top-ten authors’ names on them (No in Fig. 4) (*U* = 75,011.500, *Z* = − 9.265, *p* < 0.001, $r_{G}$ = 0.548). On the other hand, when the effect of top-ten authors on publication time was evaluated separately for single- and double-blind journals, single-blind journals stand out with the more pronounced difference ($\eta_{H}^{2}$ = 0.046) in comparison with double-blind ones ($\eta_{H}^{2}$ = 0.024).

### What if an editorial board member is also one of the top-ten authors?

It was revealed that being an editorial board member or one of the top-ten authors of a journal shortens the publication time. As the second step, we tried to answer the question of what if an editorial board member is also one of the top-ten authors? According to the results (See Fig. 5), if the editorial board members in both single and double-blind peer review were also one of the top-ten authors and submitted their articles to their journals, it took an average of 100 days to complete all processes. Having one editorial board member or one top-ten as a co-author may also shorten the process. One of the interesting findings of our study is the longest publication time (231 days) for the papers written in collaboration with top-ten authors and by editorial board members. It can be commented that editors try to avoid self-bias on publication time. However, future investigations are needed to understand the real reasons behind it.

**Fig. 6:**
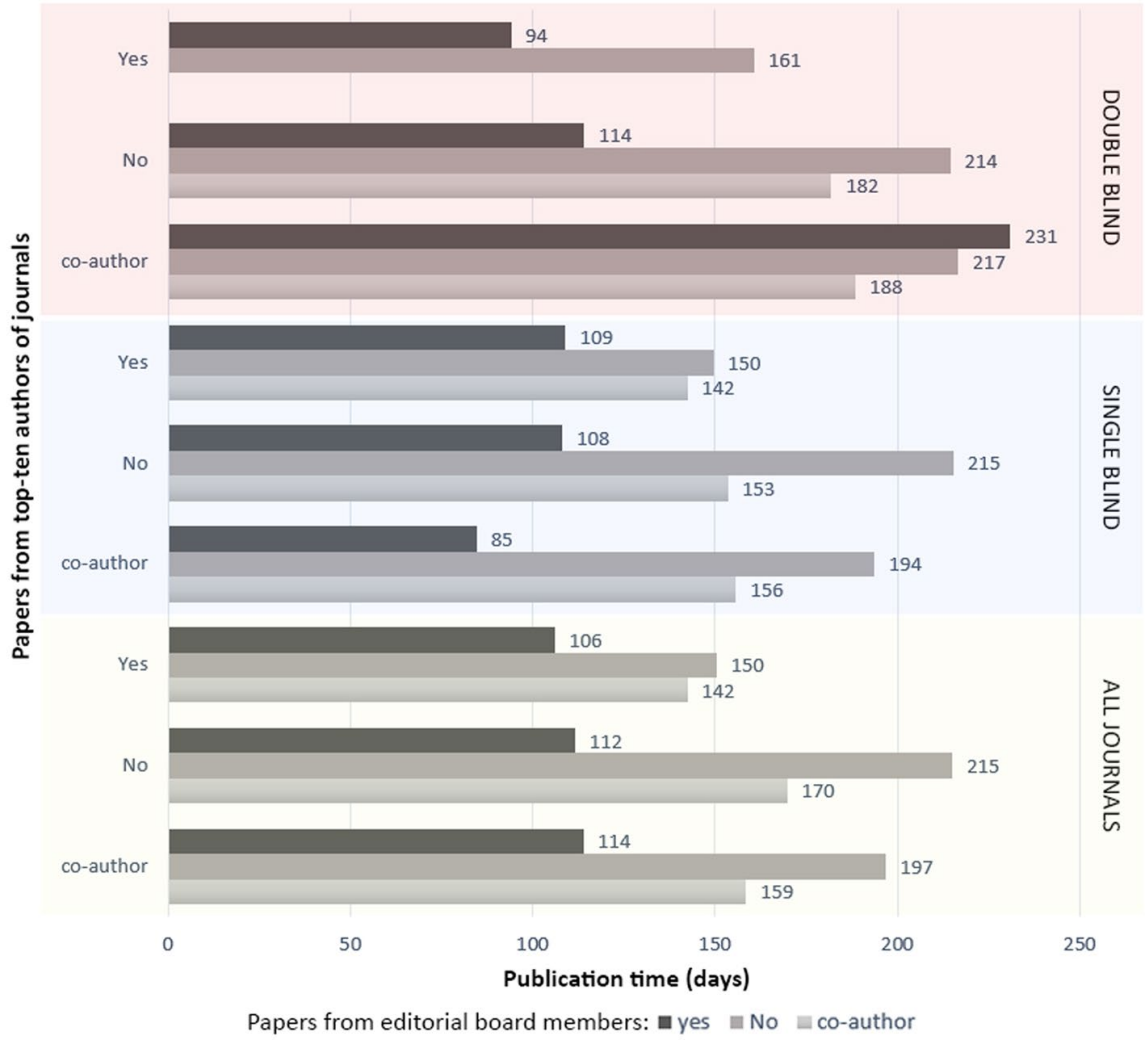
Average publication times for the papers written by editorial board members and top-ten authors

### Publication times of papers written by central researchers of the LIS field

All the findings of previous parts have proved that there are popular, successful, and experienced researchers working in the LIS field, and their papers published faster than the others, as expected. Looking from a wider perspective, a total of 6814 unique authors published 3816 papers in LIS. Some authors serve more than one journal as an editorial board member. Besides, most of the top-ten authors of single-blind journals are also editorial board members. Figure 6 shows the distribution of authors with the roles and publication speed of authors placed in the centre of the LIS network. In a section of Fig. 6, the author pool of information science field is visualised. The circle inside the clusters represents the number of core figures of the field. The b part shows the publication speed of articles written by these core authors.

**Fig. 6:**
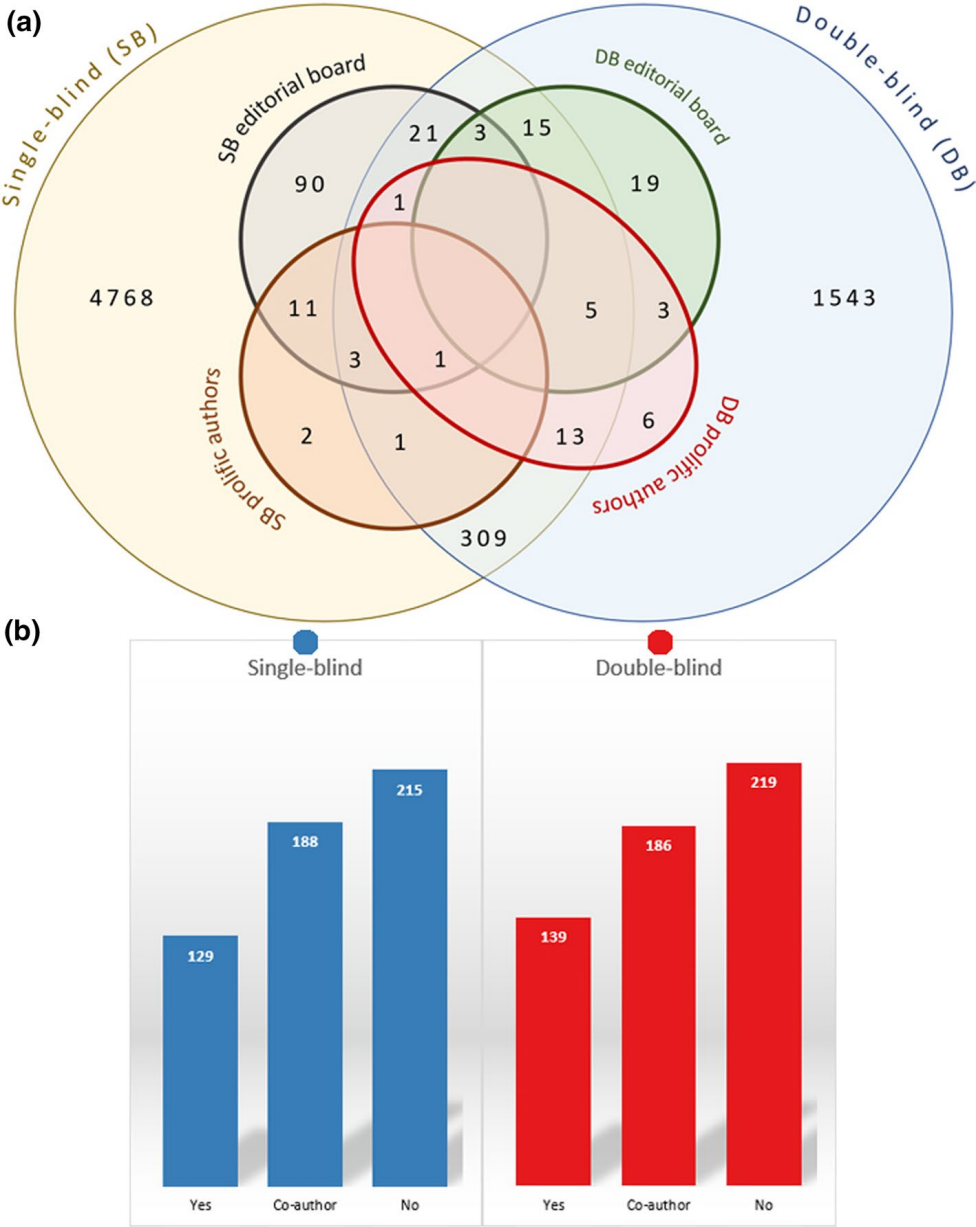
**a** Number of unique authors in LIS journals and distribution of their roles, **b** Publication speed of articles written by 194 central authors that have two or more roles in LIS

147 (3.8%) of the articles were single-authored and written by central authors, while 748 (19.6%) of them had central co-authors. Central authors’ papers were published in a short time compared to the other authors’ papers. Kruskal Wallis test confirms this finding (*H*(2) = 146.897, *p* < 0.001, $\eta_{H}^{2}$ = 0.038).

### How does the number of authors affect the publication time?

The analysis showed that the number of authors affects the publication time. Generally, as the number of authors increases, so does the publication time (see Table 1). More authors can mean more time needed for revisions and getting the approvals from all authors. Besides, it is well known that collaborative papers have higher citation potentials especially for the cases of international collaborations (e.g. Bornmann, 2017; Rousseau & Ding, 2016). Considering this fact, editors or reviewers can make positive decisions about collaborative papers. Regards our journal-based analyses, the time in double-blind journals (*ASLIB, JDOC, OIR*) did not differ by the number of authors whereas there was a significant difference for single-blind journals *(JASIST, SCIM, JOI*). The most evident difference is for *JOI*, in which the median time for single-author articles are 117 days, while it doubles for articles with 6 authors (233 days).

**Table 1:** Statistics for publication time by the number of authors

N of authors		1	2	3	4	5	6	7+	Test results
All	N	702	1114	996	541	268	110	85	*H*(6) = 111.210, *p* < 0.001, $\eta_{H}^{2}$ = 0.028
	Median	144	172	194	196	195	210	181	
*JASIST*	N	102	236	371			44		*H*(3) = 17.623, *p* = 0.001, $\eta_{H}^{2}$ = 0.019
	Median	175	208	244			256		
*SCIM*	N	275	493	847			100		*H*(3) = 33.498, *p* < 0.001, $\eta_{H}^{2}$ = 0.017
	Median	154	167	189			177		
*JOI*	N	60	102	198			15		*H*(3) = 22.197, *p* < 0.001, $\eta_{H}^{2}$ = 0.051
	Median	177	133	160			233		
*ASLIB*	N	49	69	97			10		*H*(3) = 1.756, *p* = 0.624, $\eta_{H}^{2}$ = 0.006
	Median	147	158	153			144		
*JDOC*	N	151	117	99			14		*H*(3) = 9.246, *p* = 0.026, $\eta_{H}^{2}$ = 0.017
	Median	126	141	136			102		
*OIR*	N	65	97	193			12		*H*(3) = 6.307, *p* = 0.098, $\eta_{H}^{2}$ = 0.009
	Median	245	258	279			342		

### The relation between publication time and citations after publication

Median publication times were compared in terms of citations received to see whether there is a shortening effect of citation potentials of papers. If a paper is on a popular subject or written by well-known authors, this paper is likely to be cited when it is published. Considering this, we checked the number of citations the articles received after publication. Table 2 shows publication time in terms of citation per year that is calculated for the publication year of the papers. It was found for single-blind papers that papers with higher citation per year had a shorter publication time. On the other side, this is not the case for double-blind papers. Based on the journals, no statistically significant difference was found only for *OIR*. The time differences in terms of citation per year are most evident for *JASIST,* which is a single-blind journal. Although Table 2 is based on publication year, the results are similar when citation per year is calculated in terms of acceptance year.^9^

**Table 2:** Statistics for publication time by the citations per year

Citation per year		0	0,17–2	2,17–4	4,20–7	7,20 +	Test results
Single	N	459	1224	603	342	215	*H*(4) = 82.907, *p* < 0.001, $\eta_{H}^{2}$ = 0.003
	Median	212	189	178	160	145	
Double	N	293	442	153	64	21	*H*(4) = 6.664, *p* = 0.155, $\eta_{H}^{2}$ = 0.028
	Median	159	173	177	167	142	
*JASIST*	N	96	362	158	84	53	*H*(4) = 40.360, *p* < 0.001, $\eta_{H}^{2}$ = 0.050
	Median	298	229	218	164	120	
*SCIM*	N	315	735	368	182	115	*H*(4) = 29.971, *p* < 0,001, $\eta_{H}^{2}$ = 0.016
	Median	203	176	168	169	157	
*JOI*	N	48	127	77	76	47	*H*(4) = 12.971, *p* = 0.011, $\eta_{H}^{2}$ = 0.027
	Median	185	159	147	131	116	
*ASLIB*	N	77	92	33	18	5	*H*(4) = 19.099, *p* = 0.001, $\eta_{H}^{2}$ = 0.073
	Median	184	153	153	116	127	
*JDOC*	N	124	176	61	14	6	*H*(4) = 15.245, *p* = 0.004, $\eta_{H}^{2}$ = 0.033
	Median	118	139	138	100	108	
*OIR*	N	92	174	59	32	10	*H*(4) = 4.600, *p* = 0.321, $\eta_{H}^{2}$ = 0.004
	Median	279	273	260	250	188	

### The effect of author countries on publication time

More than half of the 3816 papers are Europe & Central Asia or North America addressed (36.5% Europe & Central Asia, 14.7% North America, 3% Europe & Central Asia and North America co-authored). One of every five papers is from East Asia & Pacific. In almost 16% of the papers have at least one author from Europe & Central Asia and/or North America and one author from other country groups. Whether there was an effect of having at least one Europe & Central Asia or North America address on the paper, the publication times were compared by country groups, and a statistically significant difference was found (*H*(8) = 125.253, *p* < 0.001, $\eta_{H}^{2}$ = 0.031). Table 3 presents the number of papers and median publication times in terms of country groups. The results are similar for single (*H*(8) = 90.152, *p* < 0.001, $\eta_{H}^{2}$ = 0.029) and double (*H*(8) = 53.199, *p* < 0.001, $\eta_{H}^{2}$ = 0.047) blind papers.

**Table 3:** Publication times in terms of country groups

Country group	N	%	Median
Latin America & the Caribbean	128	3.4	208
South Asia	85	2.2	206
East Asia & Pacific	764	20.0	205
Collaborative studies of Europe & Central Asia and North America with the other groups	605	15.9	194
Collaborative studies of the other groups without Europe & Central Asia or North America	35	0.9	193
North America	562	14.7	172
Collaboration of Europe & Central Asia and North America	116	3.0	168
Middle East & North Africa	104	2.7	162
Sub-Saharan Africa	26	0.7	161
Europe & Central Asia	1391	36.5	156

Similarly, country group income affects publication time (*H*(6) = 44.249, *p* < 0.001, $\eta_{H}^{2}$ = 0.010). The number of papers and median publication times for different income groups is presented in Table 4. First of all, note that 66% of all papers are from high-income countries. Upper middle-income country addressed (16.5%), and co-authored papers of high and upper-middle-income countries (12.5%) follows. Only 98 papers (2.6%) don’t have a co-author from high and/or upper middle-income countries. As can be seen in Table 4, a co-author from a high-income country has a very positive effect on shortening the publication time.

**Table 4:** Publication times in terms of country group income

Country group income	N	%	Median
Collaboration of Upper Middle, Lower Middle- & Low-Income countries	12	0.3	246
Collaboration of Lower Middle- and Low-Income countries	98	2.6	216
Collaboration of High Income, Upper Middle Income, Lower Middle- & Low-Income countries	13	0.3	206
Collaboration of High Income and Upper Middle-Income countries	478	12.5	196
Upper Middle-Income countries	631	16.5	192
Collaboration of High Income and Lower Middle- & Low-Income countries	60	1.6	192
High Income countries	2524	66.2	170

## Discussion and conclusions

This paper seeks factors affecting the publication speed. Many reasons such as the quality of articles, the date of the article submitted, publisher’s publication practices or the workload of editors, can affect the publication time. However, in this study, we aimed to reveal whether there are researchers who have an advantage on publication speed.

According to our results, papers belonging to editorial board members and top-ten authors are published faster. The main reason beyond the apparent reason should be taken into account when evaluating this finding. Publishing fastis of course closely related to the experience these people have and the quality of the papers they submitted, beyond solely being an editorial board member and/or top-ten authors. Also, experienced authors know the publication processes and expectations of the journals and write their papers accordingly. It provides them a time advantage. In this point, one of the important findings of our study is the advantage of young researchers who work with experienced researchers. This study shows that not only experienced researchers, but also researchers who publish papers with them have the advantage of the speed of publication. This inequality is worth examining in future studies.

The editorial board members and top-ten authors have high scientific levels and qualifications, and the outputs produced by these authors have a significant scientific level. Besides, the leading figures in the fields are usually invited as editorial board members to the journals. It is expected that the papers written by prestigious authors have good quality and so, their papers are published faster than the papers written by others. However, the focus of this paper is not the quality of the papers. It aims to reveal the current practices of journals and to present the publication time differences in scholarly communication processes of articles written by known and unknown authors. Furthermore, the paper is limited to only six journals in the LIS field, but, as indicated in the Methodology part, the volumes of the journals are not the same. It means the workloads of editors are not equal. On the other hand, the audiences of the journals vary. While some journals can be considered as traditional information science journals such as *ASLIB* or *OIR*, some journals (*SCIM* or *JASIST*) have more authors from different disciplines. Lastly, all the double-blind journals in our dataset (*ASLIB*, *JDOC* and *OIR*) are published by the same publisher (Emerald). It points to a limitation that some differences found between single- and double-blind reviews might be due to peculiarities of the publisher rather than the peer review model itself. All these factors make the comparison between journals harder. Therefore, more investigations are needed to understand all the factors affecting the publication time. On the other hand, the effect of Covid-19 pandemic to publication time and shorter review time of papers dealing with the issue of Covid-19 pandemic may be subject of another papers.

We also found that the number of authors of the articles affects the publication times. It can be an expected finding since the more authors, the more time needed to revise the papers. However, the significant difference between single- and double-blind reviews is worth noting. It is important to understand the reasons behind this difference in future studies.

The other important finding is the significant difference between the number of citations and the publication times for single-blind peer-review. It can be commented that reviewers or editors tend to accept papers that have good citation potential. Future investigations are needed to understand the factors creating the relation between the number of citations and publication time.

In addition to the time advantage of some researchers, this study also shows the importance of sharing the processing dates of research in detail by the publishers. One can calculate publication time from submission to decision using the information provided by publishers, however, it is also important to understand the reasons behind editorial delays. Are the delays related to editorial issues, peer-review delay, or author revision processes? The best way to understand is to provide detailed statistics for articles. Bilalli et al. (2021) indicated that all dates of publication processes (receive, revision, acceptance, etc.) must be provided by journals. However, the information provided by journals about the review durations is limited especially for single-blind journals. Also, it is not provided as a metadata element in publishers’ databases and it requires data mining. To be able to make accurate analyses, this information should be served by publishers and added to databases.

## Data Availability

Scripts used for the study available at Ataskin/article_date [Python] https://github.com/ataskin/article_date. The data is available at http://zehrataskin.com/SCIM-ISSI-2021/raw_data_zt-at-gd-ek.csv.

## Footnotes

1 The co-occurrence network based on author keywords was created using VOSviewer. The search was done on 1st December, 2021 on Web of Science Core Collection using TS = (“time to publication” OR “publication speed” OR “publication time” OR “publication delay” OR “editorial delay” OR “publishing delay” OR “publication lag”) search term. Metadata of 696 articles downloaded.

2 https://www.elsevier.com/journals/journal-of-informetrics/1751-1577/guide-for-authors.

3 https://asistdl.onlinelibrary.wiley.com/hub/journal/23301643/homepage/forauthors.

4 https://www.springer.com/journal/11192/submission-guidelines.

5 https://www.emeraldgrouppublishing.com/journal/ajim#author-guidelines.

6 https://www.emeraldgrouppublishing.com/journal/jd#author-guidelines.

7 https://www.emeraldgrouppublishing.com/journal/oir#author-guidelines.

8 The difference is significant for all the pairs. According the effect sizes calculated, the most significant difference is between the papers of editors (Yes in Fig. 3) and papers with no editors (No in Fig. 3) (*U* = 106,418.000, *Z* = − 7.636, *p* < 0.001, $r_{G}$=0.420).

9 For single blind papers: *H*(4) = 93.354, *p* < 0.001, $\eta_{H}^{2}$ = 0.032; For double blind papers: *H*(4) = 8.394, *p* = 0.078, $\eta_{H}^{2}$ = 0.017; For *JASIST*: *H*(4) = 45.775, *p* < 0.001, $\eta_{H}^{2}$ = 0.057; For *SCIM*: *H*(4) = 28.896, *p* < 0.001, $\eta_{H}^{2}$ = 0.015; For *JOI*: *H*(4) = 14.744, *p* = 0.005, $\eta_{H}^{2}$ = 0.032; For *ASLIB*: *H*(4) = 16.547, *p* = 0.002, $\eta_{H}^{2}$ = 0.061; For *JDOC*: *H*(4) = 11.294, *p* = 0.023, $\eta_{H}^{2}$ = 0.022; For *OIR*: *H*(4) = 7.929, *p* = 0.094, $\eta_{H}^{2}$ = 0.014.
